# Characteristics, comparative genomics, and phylogenetic analysis of the chloroplast genome of *Rosaplatyacantha*

**DOI:** 10.7717/peerj.21639

**Published:** 2026-08-17

**Authors:** Gang Lu, Mengmeng Yu, Fazu Xu, Youju Wang, Xiaoqing Zhang, Tingliang Xu, Fashou Liu, Yihui Liu

**Affiliations:** 1 Key Laboratory of Qinghai Province for Landscape Plants Research, Plateau Flower Research Centre, College of Agriculture and Animal Husbandry, Qinghai University, Xining, China; 2 Medical College of Qinghai University, Xining, China; 3 Botanical Garden of Datong County, Datong County, Xining, China; 4 Laboratory for Research and Utilization of Qinghai Tibet Plateau Germplasm Resources, Academy of Agriculture and Forestry Sciences of Qinghai University, Xining, China

**Keywords:** *Rosa platyacantha*, Chloroplast genome, Comparative genomics, Phylogeny

## Abstract

**Background:**

*Rosa platyacantha* is a rose species endemic to the high-altitude regions of Xinjiang in China. To date, no complete chloroplast genome has been reported for this species, limiting our understanding of its genomic characteristics and phylogenetic relationships within the genus *Rosa*.

**Methods:**

This study employed Sequencing by Synthesis (SBS) technology to achieve the first complete sequencing, assembly, and annotation of the *R. platyacantha* chloroplast genome. Comparative genomics and phylogeny analyses were then conducted using this data alongside chloroplast genome data from other *Rosa* species.

**Results:**

The chloroplast genome of *R. platyacantha* spans 157,133 bp, and it annotates 132 genes and has a total guanine cytosine (GC) content of 37.21%. Fifty-one simple sequence repeat (SSR) loci were identified, predominantly adenine/thymine (A/T)-type mononucleotide repeats. Codon usage preference analysis revealed a marked bias towards synonymous codons ending in adenine/uracil (A/U). Compared to closely related species, *R. platyacantha* exhibited higher nucleotide diversity (Pi) in non-coding regions and the large single copy (LSC) and small single copy (SSC) region. Potential adaptive hot spots were identified at ycf3-trnS -GCU (Pi = 0.29005), *trnC*-GCA, *trnT*-UGU, and *trnV*-UAC. Rose genus chloroplast genomes are generally conserved, with most species exhibiting identical inverted repeat regions a and b (IRa and IRb) lengths. However, only *R. acicularis* shows a 62 bp difference, and the associated fragment length of the duplicated gene *ycf1* varies between species from 1,108 to 1,117 bp. Phylogeny analysis revealed that *R. platyacantha* did not cluster with the sympatric *R. fedtschenkoana*. This finding confirms their distant evolutionary relationship at the genome level, providing new insights into the evolutionary and dispersal pathways of *Rosa* species within the unique habitats of northwest China.

## Introduction

The genus *Rosa* L., which belongs to the Rosaceae family, comprises over 200 species worldwide. Of these, 95 are distributed across China, including 65 endemic species. They are widely distributed throughout temperate and subtropical regions of the Northern Hemisphere (Yang, Wei & Ge, 2016). The false fruit of *Rosa* species, known as rose hips, has a fleshy texture and is rich in diverse nutrients. It is classified as a fruit with both medicinal and edible properties (Gudarzi et al., 2024; Wang et al., 2023). Furthermore, rose hips are a natural source of antioxidants and contain bioactive compounds such as polyphenols, terpenoids and ascorbic acid (Zhou et al., 2024b; Zhou et al., 2023). Phenolic acids are particularly significant as the human body cannot synthesize them and must obtain them through the consumption of fruits, vegetables, and other foods (Shi et al., 2024). Rose hips exhibit antioxidant, anti-inflammatory, antibacterial and anti-ageing properties, as well as diverse pharmacological effects including anti-cancer, anti-diabetic, cardiovascular disease prevention and anti-ulcer activities (Li et al., 2024). *Rosa platyacantha*, a primary variety within the *Rosa* genus, is predominantly found in China’s Xinjiang region. Research indicates that it contains flavonoids, phenolic compounds, and triterpenoids, which exhibit anti-ageing, antioxidant, and immune-enhancing properties (Wang et al., 2023; Zhou et al., 2024b).

However, species within the genus *Rosa* exhibit a high degree of morphological similarity in their floral, foliage, and thorny structures—both vegetative and reproductive organs. Coupled with their highly heterozygous genomes, natural interspecific hybridisation and artificial hybridisation frequently occur, yielding fertile offspring. This has resulted in a proliferation of hybrid varieties on the market, posing significant challenges for the classical taxonomic identification of *Rosa*’s original species (Zhang et al., 2024). Despite attempts by researchers to clarify the taxonomic system through methods combining cytogenetics and molecular markers, a widely accepted phylogeny framework remains elusive to this day (Zhao et al., 2023). Comprehensive molecular phylogeny and evolutionary biological studies on *Rosa* species classification are lacking, with only a few studies employing short gene fragments for species identification (Schanzer, Fedorova & Meschersky, 2024). In most angiosperms, the chloroplast genome (cpDNA) follows a maternal inheritance pattern, with a minority exhibiting biparental inheritance (Kim et al., 2024). Stable chloroplast genomes provide relatively reliable molecular phylogeny analysis for species identification, resolving taxonomic controversies and reconstructing phylogeny lineages (Qin et al., 2025). The structure of angiosperm chloroplast genomes exhibits a typical quadripartite organisation, typically comprising a large single-copy region (LSC), a small single-copy region (SSC), and two inverted repeat regions (IRa and IRb). The genome length ranges from 120 to 160 kb and contains approximately 130 genes (Liu et al., 2022a). The chloroplast genome is a valuable tool for assessing genetic diversity, tracing maternal lineages of hybrids, elucidating evolutionary origins and conducting Phylogeny analyses. It provides crucial insights into complex evolutionary mechanisms (Daniell et al., 2016). In recent years, the sequencing and assembly of chloroplast genomes from *Rosa* species has progressively revealed their advantages; however, the limited number of reported species restricts our understanding of the phylogeny relationships within this genus (Liu, He & Qiu, 2022b; Yang, 2019; Zhou et al., 2025).

This study involved the complete sequencing, assembly and annotation of the *R. platyacantha* chloroplast genome. A systematic investigation will be conducted across three dimensions: the structural characteristics of the chloroplast genome, comparative genome analysis, and phylogeny. The molecular properties of the *R. platyacantha* chloroplast genome, including its structural features, repetitive sequence distribution, codon bias and nucleotide polymorphism, will be analysed systematically. Through comparative analysis with closely related species, the study examined the expansion/contraction dynamics of the intercalary region (IR), genome sequence differentiation and visualisation characteristics in a systematic manner. Furthermore, a maximum likelihood (ML) phylogeny tree was reconstructed to elucidate evolutionary relationships within the genus *Rosa*. It also provides the theoretical basis for the subsequent studies of *Rosa* on the origin, affinity, and phylogeny and helps with the rational conservation and development of the excellent germplasm.

## Materials and Methods

### Plant material collection, DNA extraction and sequencing

Leaf tissue samples were collected from a single representative individual of *R. platyacantha* growing in the Aheya River valley region of Kaxiagar Town, Zhaosu County, Ili Kazakh Autonomous Prefecture, Xinjiang Uygur Autonomous Region, China (81°00′E, 42°45′N). Sufficient quantities of fresh, healthy leaves were collected and placed in self-sealing bags containing silica gel. Upon returning to the laboratory, the leaves were rinsed with grade 1 water, dried, transferred into centrifuge tubes, labelled and stored in liquid nitrogen for future use. genome DNA from *R. platyacantha* was extracted using a modified CTAB method (Yan et al., 2008), after which it was sent to Kangsheng Xuyuan Biotechnology Co., Ltd. (Wuhan, China) for sequencing. genome DNA samples were analysed comprehensively *via* agarose gel electrophoresis and Nanodrop detection. The concentration of the DNA was then quantified precisely using a Qubit 3.0. Following successful validation of the samples, the DNA was randomly shared using a Covaris ultrasonic homogeniser. The library preparation workflow then continued with terminal repair, A-tailing, adapter ligation, purification and PCR amplification. Once the library was complete, an initial quantification was performed using a Qubit 3.0, followed by library dilution. The insert size was then assessed using a Qsep100. Once the inserts met the required specifications, the library’s effective concentration was precisely quantified *via* quantitative polymerase chain reaction (Q-PCR) (effective concentration >3 nM) to ensure quality.After successful quality control of the libraries, they were pooled onto flow cells according to the required effective concentrations and target sequencing data volumes. After cBOT clustering, the Illumina high-throughput platform (HiSeq X) was used for sequencing. The Illumina HiSeq platform uses Sequencing by Synthesis (SBS) technology to sequence DNA libraries. The sequencing data underwent quality control using FastQC (http://www.bioinformatics.babraham.ac.uk/projects/fastqc), yielding high-quality data with a quality score of at least 99.63%.

### Chloroplast genome assembly and annotation

The optimised sequences were assembled using multiple k-mer parameters with the online assembler SPAdes v3.11.1 (Bankevich et al., 2012). The *R. platyacantha* genome sequence was extracted using Bowtie software (Langmead, 2009), followed by assembly with SPAdes. Gene annotation was performed using DOGMA and CPGAVAS software (Liu et al., 2012; Wyman, Jansen & Boore, 2004). The genome map was visualised using OGDRAW (Marc et al., 2013). The raw sequencing data has been uploaded to the NCBI database (PZ127297).

### Repeat sequence analysis

Simple sequence repeat (SSR) (microsatellite) sequences within the chloroplast genome were identified using the MISA v2.1 online tool (https://webblast.ipk-gatersleben.de/misa/) (Beier et al., 2017). The following parameters were used to detect repeat sequences: mononucleotide repeats ≥10 times, dinucleotide repeats ≥6 times, trinucleotide repeats ≥5 times, tetranucleotide repeats ≥5 times, pentanucleotide repeats ≥4 times and hexanucleotide repeats ≥4 times.

### Analysis of codon usage

CodonW software (version 1.4.2; http://sourceforge.net/projects/codonw/) was used to calculate the relative synonymous codon usage (RSCU) values (Jia et al., 2025). RSCU is defined as the ratio of the actual frequency of codon usage to the expected frequency if all codons were used equally. An RSCU value of 1.0 indicates no usage preference; values below 1.0 indicate relatively low usage frequency for that codon; and values above 1.0 indicate relatively high usage frequency.

### Comparative genome analysis

#### Whole-genome alignment and visualisation

In order to investigate evolutionary relationships, complete chloroplast genome sequences for 10 *Rosa* species were downloaded from the NCBI database. *R. fedtschenkoana* (RF, MZ_261857); *R. kokanica* (RK, MW_298478); *R. hybrid* cultivar (RH1, MZ_261860); *R. rugosa* × *R. sertata* (RR, MT_845214); *R. sertata* (RS, NC_066975); *R. hybrid* cultivar (RH2, MZ_261892); *R. minutifolia* (RM1, MT_755634); and *R. palustris* (RP, MZ_261868); *R. acicularis* (RA, NC_068090)*.* A comparative analysis of genome structure and sequence composition was conducted between *R. platyacantha* and the selected closely related species. Whole-genome sequence divergence was visualised using the mVISTA online tool (https://genome.lbl.gov/vista) to assess similarities and differences between genome sequences (Frazer et al., 2004).

#### Nucleotide polymorphism (Pi)

Nucleotide polymorphism was calculated using the sliding window analysis method with DnaSP v6 and VCFtools software (Librado & Rozas, 2009). The analysis covered the complete chloroplast genomes of ten related species, and statistical significance was assessed *via* a permutation test. The software parameters were set as follows:Window length: 600 bp, Step size: 200 bp, *X*-axis: midpoint position within the window. *Y*-axis: nucleotide diversity within each window.

#### IR region boundary analysis

IRscope (https://irscope.shinyapps.io/irapp/) (Amiryousefi, Hyvönen & Poczai, 2018), an online tool, was used to analyse and visualise contraction and expansion patterns at the junctions between the LSC, SSC and IR regions in *R. platyacantha* and its closely related species.

### Phylogeny analysis

Whole-genome multiple sequence alignment was performed using the MAFFT v7.490 software combined with the FFT-NS-2 algorithm (Katoh & Standley, 2013). The ModelFinder module within IQ-TREE was used to determine the optimal nucleotide substitution model *via* Bayesian Information Criterion (BIC) screening (Kalyaanamoorthy et al., 2017). This identified the GTR+G+I model as the preferred option. Low-quality alignment regions were then filtered from the multiple sequence alignment using the trimAl v1.4.rev22 software with the ‘gappyout’ parameter enabled to retain only high-quality conserved blocks. A phylogeny tree was reconstructed using IQ-TREE v2.1.4 under maximum likelihood methodology (Minh et al., 2020). ModelFinder was then rerun on the pruned alignment to identify the optimal fit *via* the Akaike information criterion (AIC), determining the K3Pu+F+I+R3 model. Branch support was assessed through 1,000 ultra-fast bootstrap approximations (UFBoot), which enabled the reliable reconstruction of evolutionary relationships among *R. platyacantha* and its closely related congeneric species.

## Results

### Characteristics of the chloroplast genome of *R. platyacantha*

**Figure 1:**
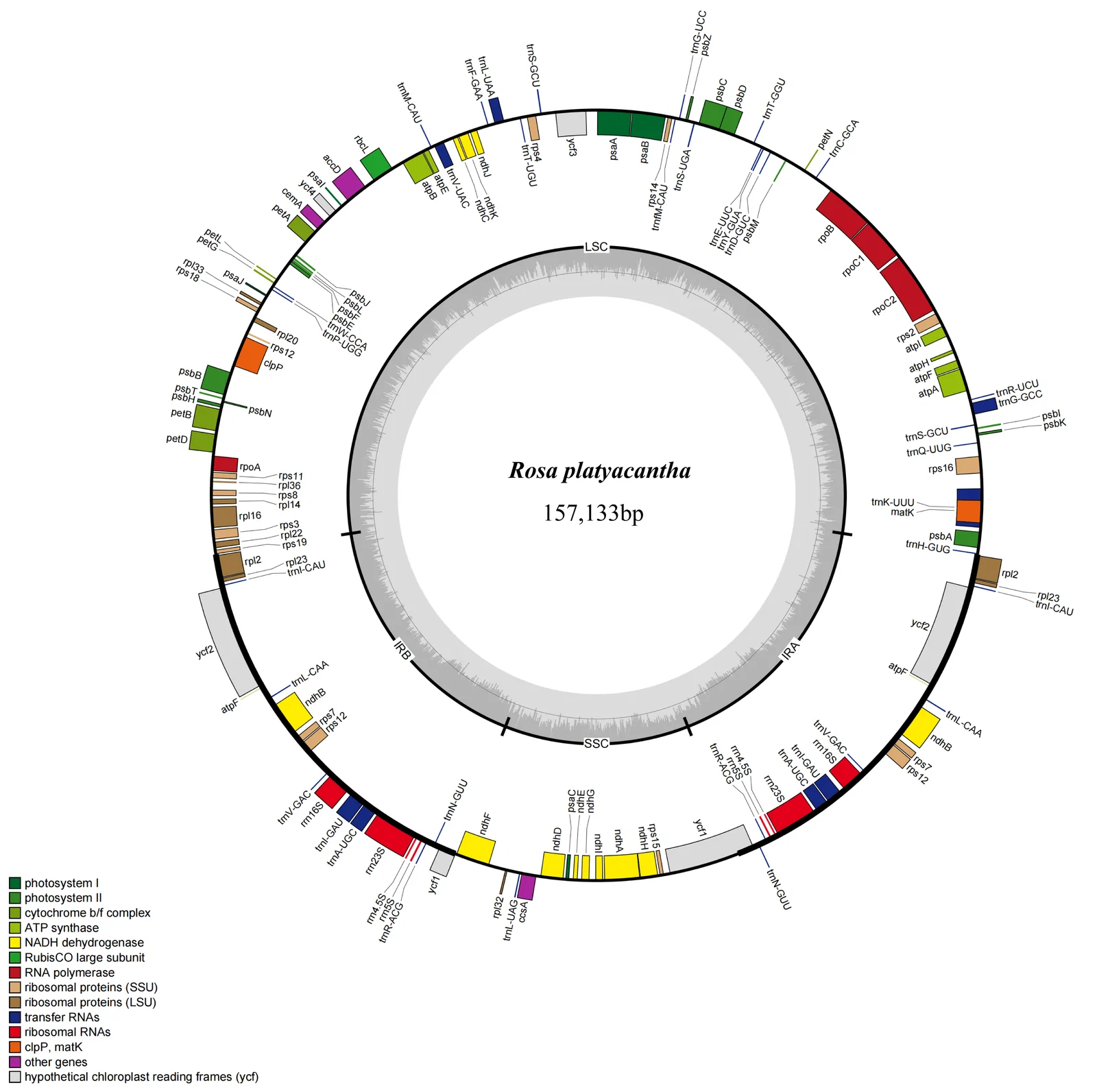
Chloroplast genome map of *R. platyacantha*. The outer-ring inner genes transcribe clockwise, and the outer-ring outer genes transcribe counterclockwise. Genes belonging to different functional categories are labelled in different colors. The inner circle shows the LSC, SSC, and IR regions. The dark grey area within the inner circle corresponds to GC content, and the light grey area to AT content.

**Figure 2:**
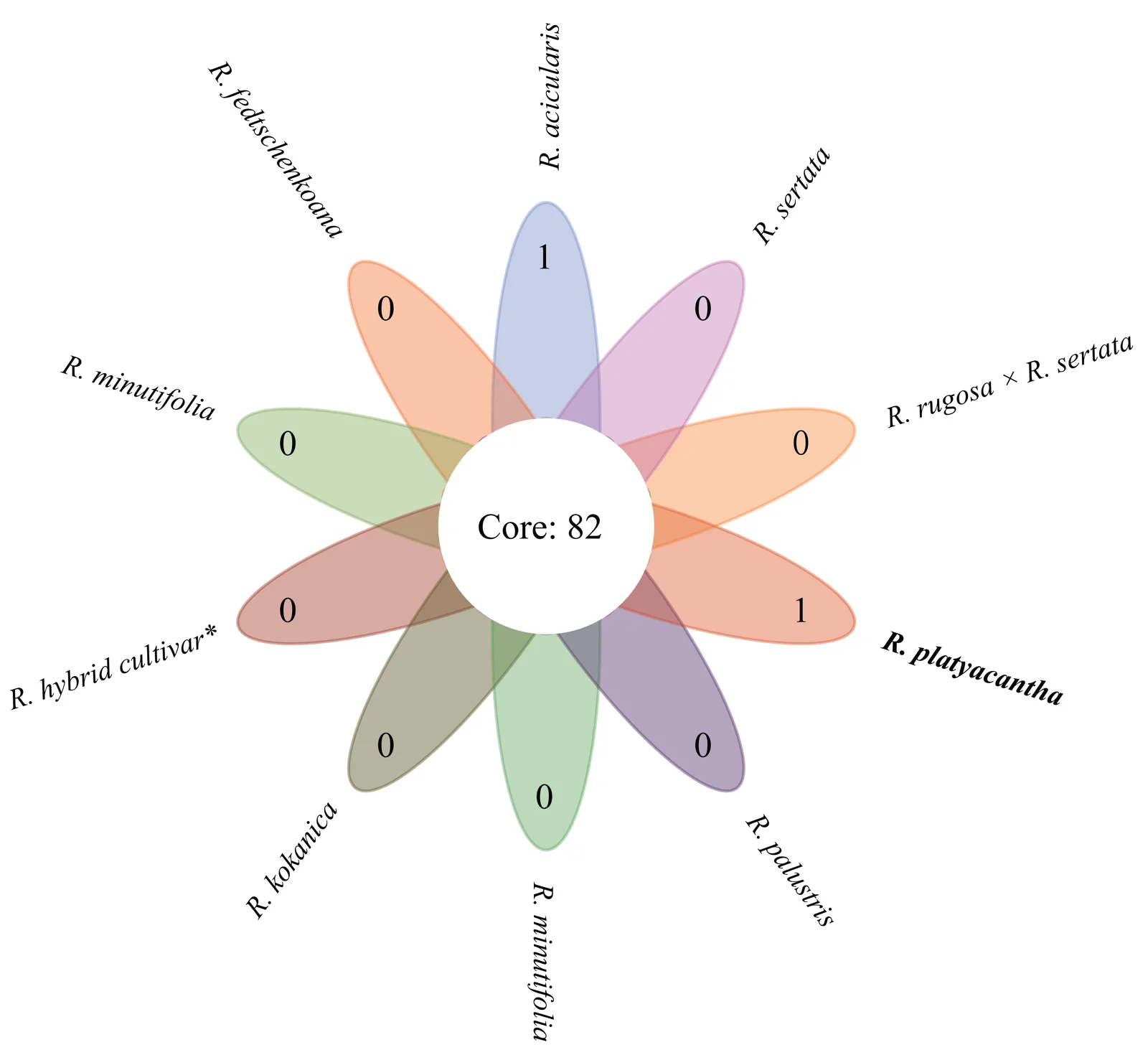
Venn diagram of shared and specific orthogonal coding genes among 10 *Rosa* species. The species names are positioned on the outermost ring, with the petals representing genetic differences. ‘0’ indicates the absence of genetic variation for that gene, while ‘1’ denotes the presence of one genetic difference. The central region (Core: 82) represents the 82 orthologous genes that are shared by all ten species.

The chloroplast genome of *R. platyacantha* exhibits a typical circular quadripartite structure (Fig. 1, Table 1), comprising one large single-copy region (LSC, 86,252 bp), one small single-copy region (SSC, 18,783 bp), and one pair of inverted repeat regions (IRs, 26,049 bp), with a total length of 157,133 bp. The overall GC content of the genome is 37.21%, with the highest GC content observed in the IR region (42.75%), followed by the LSC region (35.18%), and the SSC region (31.18%). A total of 132 genes were annotated across the genome, comprising 86 protein-coding genes, 38 tRNA genes, and eight rRNA genes. Orthologous protein-coding gene analysis (Fig. 2) revealed that 82 protein-coding genes are shared among 10 species. The *infA* gene is absent in *R. platyacantha* and *R. majalis*, the *atpF* gene is detected only in *R. platyacantha*, and the *ycf15* gene is unique to *R. acicularis*.

**Table 1:** Information on the chloroplast genome of *R. platyacantha*.

Category	Length/bp	GC/%	Number of genes
Genome	157,133	37.21	133
LSC	86,252	35.18	–
SSC	18,783	31.18	–
IR	26,049	42.75	–
CDS Genes	79,760	37.97	88
rRNA Genes	9,054	55.35	8
tRNA Genes	2,848	53.2	37
Intron	18,458	37.24	–

### Functional gene classification and intron analysis

Genes within the genome can be categorised into four groups based on their function: Photosynthetic genes (47 genes), Self-replicating genes (29 genes), Other genes (five genes) and Genes of unknown function (five genes) (Table 2). Eighteen genes are duplicated in the intergenic region, with two copies each, while the protein-coding gene *atpF* has three copies. Twenty genes collectively contain 22 introns: eight tRNA genes and 12 protein-coding genes each harbour one intron, while the protein-coding genes *clpP* and *ycf3* contain two introns each.

**Table 2:** Number of genes in the chloroplast genome of *R. platyacantha*.

Category for gene	Group of genes	Name of genes
Genes for photosynthesis	Subunits of ATP synthase	*atpA, atpB, atpE, atpF(*×3*), atpH, atpI*
	Subunits of photosystem I	*psaA, psaB, psaC, psaI, psaJ*
	Subunits of photosystem II	*psbA, psbB, psbC, psbD, psbE, psbF, psbI, psbJ, psbK, psbL, psbM, psbN, psbT, psbZ*
	Subunits of NADH-dehydrogenase	ndhA^*^, ndhB^**^(×2*), ndhC, ndhD, ndhE, ndhF, ndhG, ndhH, ndhI, ndhJ, ndhK*
	Subunits of cytochrome b/f complex	petA, petB^*^, petD^*^, petG, petL, petN
	Subunit of rubisco	*rbcL*
Self-replication	tRNA genes	trnA-UGC^**^(×2), trnC-GCA, trnD-GUC, trnE-UUC, trnF-GAA, trnfM-CAU, trnG-GCC^*^, trnG-UCC, trnH-GUG, trnI-CAU(×2), trnI-GAU^**^(×2), trnK-UUU^*^, trnL-CAA(×2), trnL-UAA^*^, trnL-UAG, trnM-CAU, trnN-GUU(×2*), trnP-UGG, trnQ-UUG, trnR-ACG(*×2*), trnR-UCU, trnS-GCU(*×2), trnS-UGA, trnT-GGU, trnT-UGU, trnV-GAC^*^(×2*), trnV-UAC, trnW-CCA, trnY-GUA*
	rRNA genes	*rrn4.5S(*×2*), rrn5S(*×2*), rrn16S(*×2*), rrn23S(*×2*)*
	Large subunit of ribosome	rpl14, rpl16^*^, rpl2^**^(×2*), rpl20, rpl22,rpl23(*×2*), rpl32, rpl33, rpl36*
	Small subunit of ribosome	*rps2, rps3, rps4, rps7(*×2*), rps8, rps11, rps12(*×2), rps14, rps15, rps16^*^, rps18, rps19
	DNA dependent RNA polymerase	rpoA, rpoB, rpoC1^*^, rpoC2
Other genes	Subunit of Acetyl-CoA-carboxylase	*accD*
	c-type cytochrom synthesis gene	*ccsA*
	Envelop membrane protein	*cemA*
	Protease	clpP^**^
	Maturase	*matK*
Genes of unknown function	Conserved open reading frames	*ycf1(*×2*), ycf2(*×2), ycf3^**^, ycf4

**Notes.**

×2 indicates 2 copy genes, ×3 indicates 3 copy genes.

* indicates a gene containing 1 intron.

** indicates a gene containing 2 introns.

### Analysis of SSR sites in the chloroplast genome

A total of 51 SSR sites were identified in the *R. platyacantha* chloroplast genome (Table 3). Of these, 47 sites (92.16%) were single-nucleotide SSRs, predominantly of the A/T base type. These were followed by dinucleotide SSRs (three sites, 5.88%) and tetranucleotide SSRs (one site, 1.96%). No trinucleotide, pentanucleotide, or hexanucleotide SSRs were detected.

**Table 3:** Frequency distribution of SSR loci in the chloroplast genome of *R. platyacantha*.

SSR Repeats	5	6	7	8	9	10	11	12	13	14	15	16	17	18	Total
A/T	–	–	–	–	–	18	13	4	2	2	2	2		1	44
C/G	–	–	–	–	–		1	1	1						3
AT/AT	–	1	1				1								3
AAAT/ATTT	1														1

### Analysis of codon usage preferences

**Figure 3:**
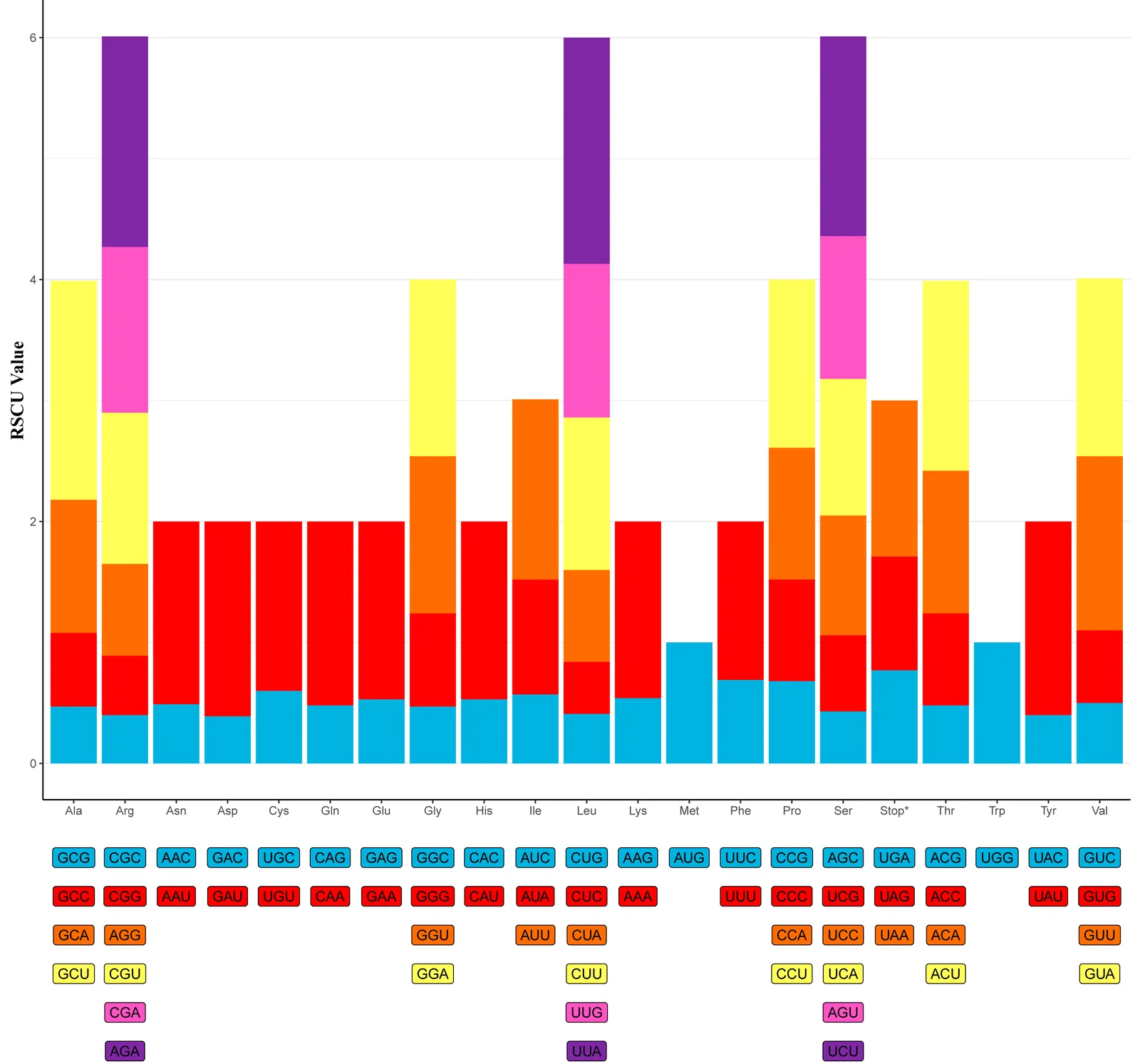
Preferred codons of the chloroplast genome of *R. platyacantha*.

Codon usage preferences were calculated based on the sequences of 61 protein-coding genes (Table 4, Fig. 3). A total of 49,638 codons were identified in all protein-coding genes (excluding the 379 stop codons). Methionine (Met, AUG) and tryptophan (Trp, UGG) are each encoded by a single codon. All other amino acids are encoded by two to six synonymous codons. The relative synonymous codon usage (RSCU) values ranged from 0.39 (the aspartic acid codon GAC) to 1.87 (the leucine codon UUA). Leucine codons exhibited the highest frequency (5,186 occurrences; 10.45%), followed by isoleucine codons (4,167 occurrences; 8.39%). Cysteine (Cys) codons displayed the lowest frequency (618 occurrences; 1.25%). Analysis of the RSCU values revealed 29 codons with RSCU > 1 (indicating a preference), 29 codons with RSCU < 1 (indicating underutilisation) and three codons with RSCU = 1 (indicating no preference). Of the 29 preferred codons (RSCU > 1), 51.72% terminated with U and 41.38% with A, indicating a significant preference for A- or U-terminating codons within the chloroplast genome of *R. platyacantha*. Furthermore, UAA was the most frequently utilised stop codon, and the preference for A/U-terminating codons exhibited a high degree of consistency across the genome.

**Table 4:** Codon analysis of the chloroplast genome of *R. platyacantha*. Asterisks (*) indicate stop codons, which do not encode any amino acid and serve as termination signals. They have been removed from the table as they are not essential for the data.

Amino acids	Codons	Count	RSCU		Amino acids	Codons	Count	RSCU
Ala	GCA	698	1.1		Pro	CCA	564	1.09
Ala	GCC	386	0.61		Pro	CCC	434	0.84
Ala	GCG	299	0.47		Pro	CCG	349	0.68
Ala	GCU	1,147	1.81		Pro	CCU	714	1.39
Cys	UGC	185	0.6		Gln	CAA	1,370	1.52
Cys	UGU	433	1.4		Gln	CAG	437	0.48
Asp	GAC	387	0.39		Arg	AGA	871	1.74
Asp	GAU	1,619	1.61		Arg	AGG	378	0.76
Glu	GAA	1,949	1.47		Arg	CGA	684	1.37
Glu	GAG	702	0.53		Arg	CGC	199	0.4
Phe	UUC	1,032	0.69		Arg	CGG	244	0.49
Phe	UUU	1,939	1.31		Arg	CGU	626	1.25
Gly	GGA	1,178	1.46		Ser	AGC	275	0.43
Gly	GGC	378	0.47		Ser	AGU	766	1.18
Gly	GGG	621	0.77		Ser	UCA	729	1.13
Gly	GGU	1,049	1.3		Ser	UCC	641	0.99
His	CAC	323	0.53		Ser	UCG	405	0.63
His	CAU	895	1.47		Ser	UCU	1,065	1.65
Ile	AUA	1,314	0.95		Thr	ACA	740	1.18
Ile	AUC	787	0.57		Thr	ACC	475	0.76
Ile	AUU	2,066	1.49		Thr	ACG	301	0.48
Lys	AAA	2,000	1.46		Thr	ACU	984	1.57
Lys	AAG	743	0.54		Val	GUA	975	1.47
Leu	CUA	655	0.76		Val	GUC	329	0.5
Leu	CUC	375	0.43		Val	GUG	399	0.6
Leu	CUG	355	0.41		Val	GUU	954	1.44
Leu	CUU	1,085	1.26		Trp	UGG	915	1
Leu	UUA	1,615	1.87		Tyr	UAC	376	0.4
Leu	UUG	1,101	1.27		Tyr	UAU	1,489	1.6
Met	AUG	1,155	1		Stop	UAA	163	1.29
Asn	AAC	606	0.49		Stop	UAG	119	0.94
Asn	AAU	1,873	1.51		Stop	UGA	97	0.77

### Comparative analysis of chloroplast genome nucleotide polymorphisms

**Figure 4:**
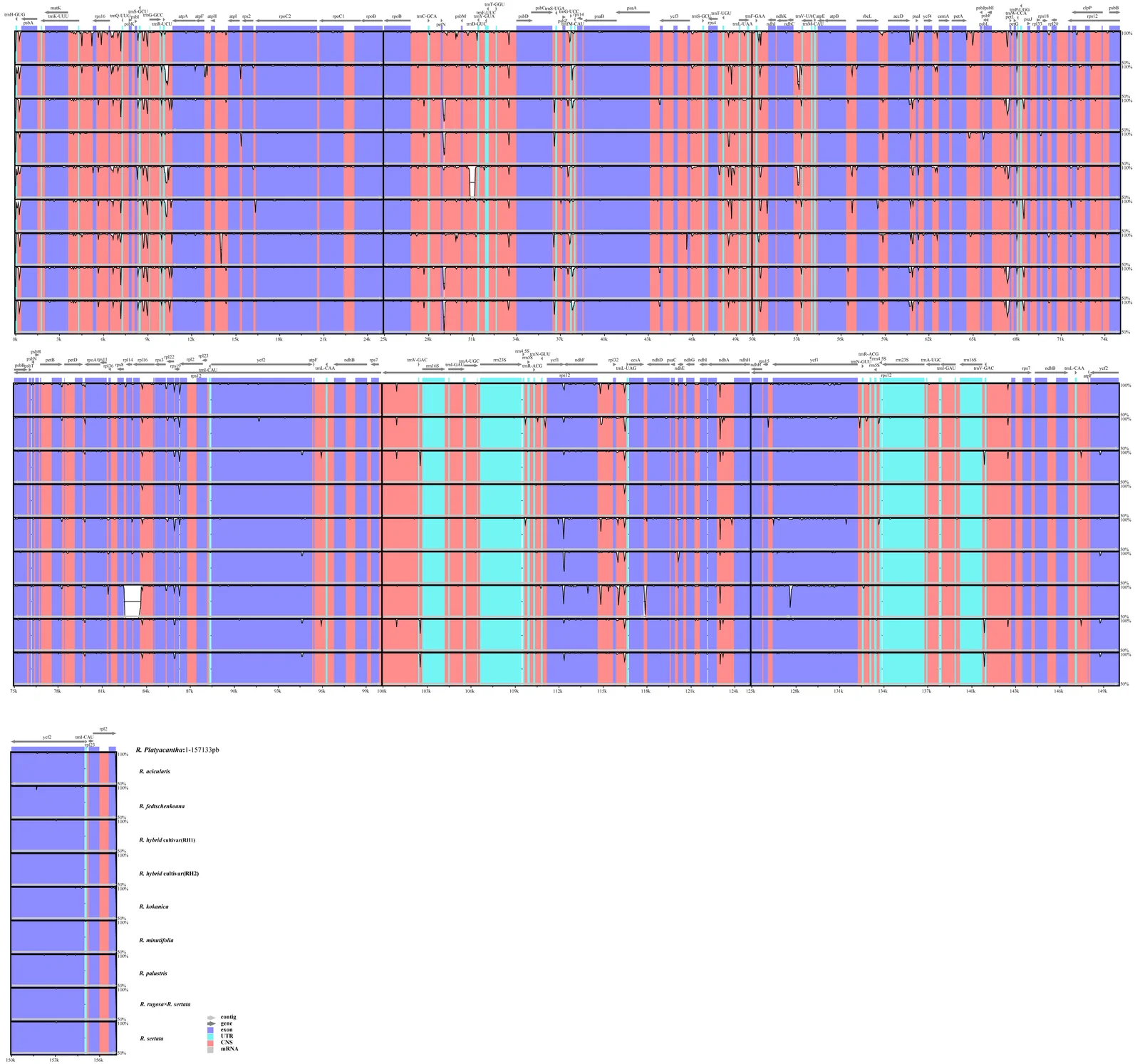
Comparison of sequence difference sites in chloroplast genomes of *Rosa*.

**Figure 5:**
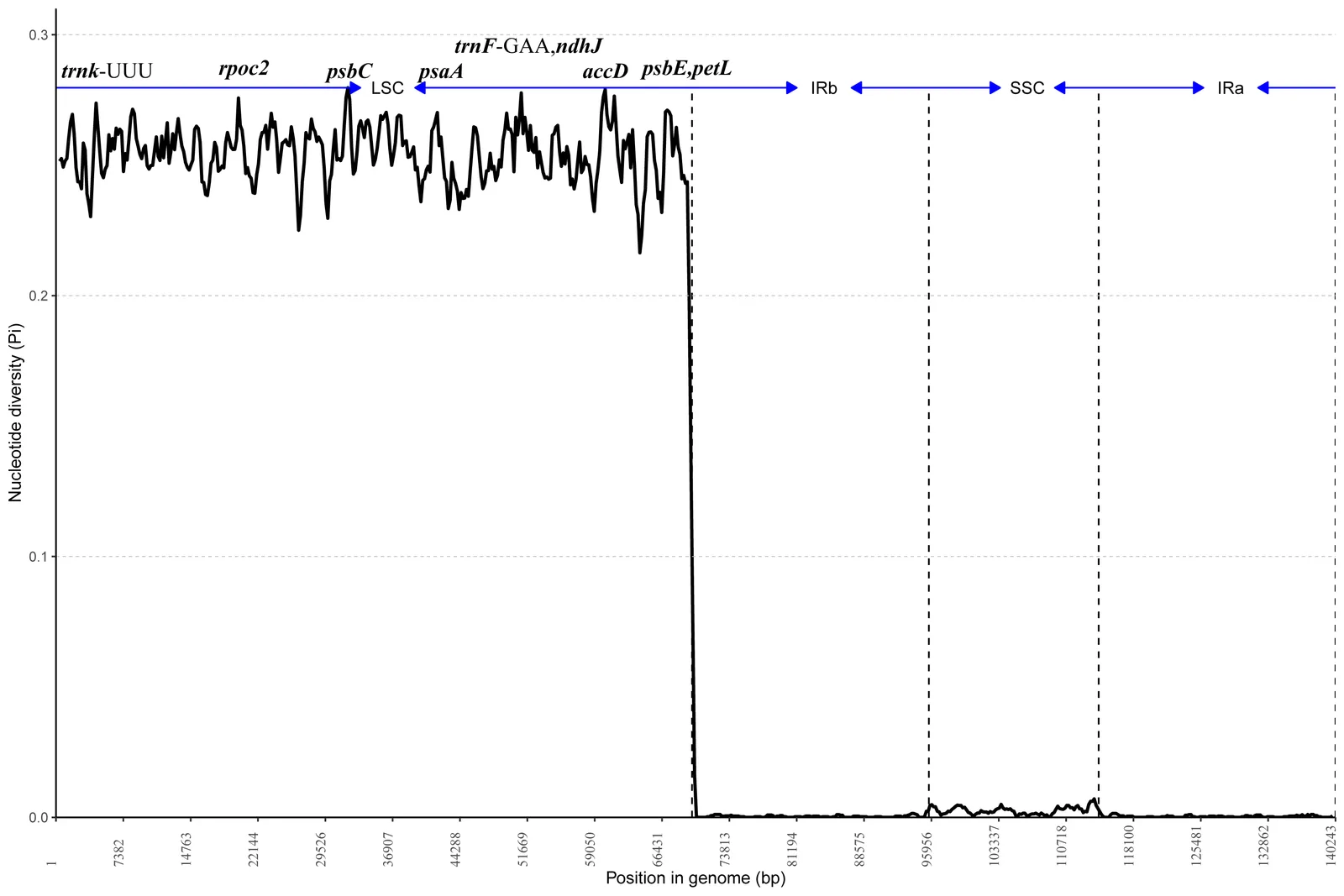
Comparison of nucleotide polymorphism (Pi) in the chloroplast genome of *R. platyacantha*.

The mVISTA software was used to compare the homology of the whole-genome sequences of the remaining 10 *Rosa* species with the reference sequence of *R. platyacantha* (Fig. 4). The results showed that the chloroplast genomes of these 10 *Rosa* species had relatively consistent sequence arrangements and were highly similar, although the ten sequences diverged to varying degrees. This was primarily evident in significantly higher sequence variation within non-coding regions than in coding regions. The Pi values for the chloroplast genome sequences of these 10 *Rosa* species were then calculated using DnaSP V6 software (Fig. 5). The results revealed that the LSC region exhibited the highest variability within the chloroplast genome, followed by the SSC region, which exhibited moderate variability. In contrast, the IR region demonstrated the lowest variability and the highest level of conservation, which is consistent with the findings in Fig. 4. Screening for highly variable regions based on nucleotide diversity identified the following hotspots: ycf3-trnS-GCU (Pi = 0.29005), trnC-GCA, trnT-UGU and trnV-UAC. All of these highly variable regions are located within the LSC region.

### Analysis of IR region boundaries

**Figure 6:**
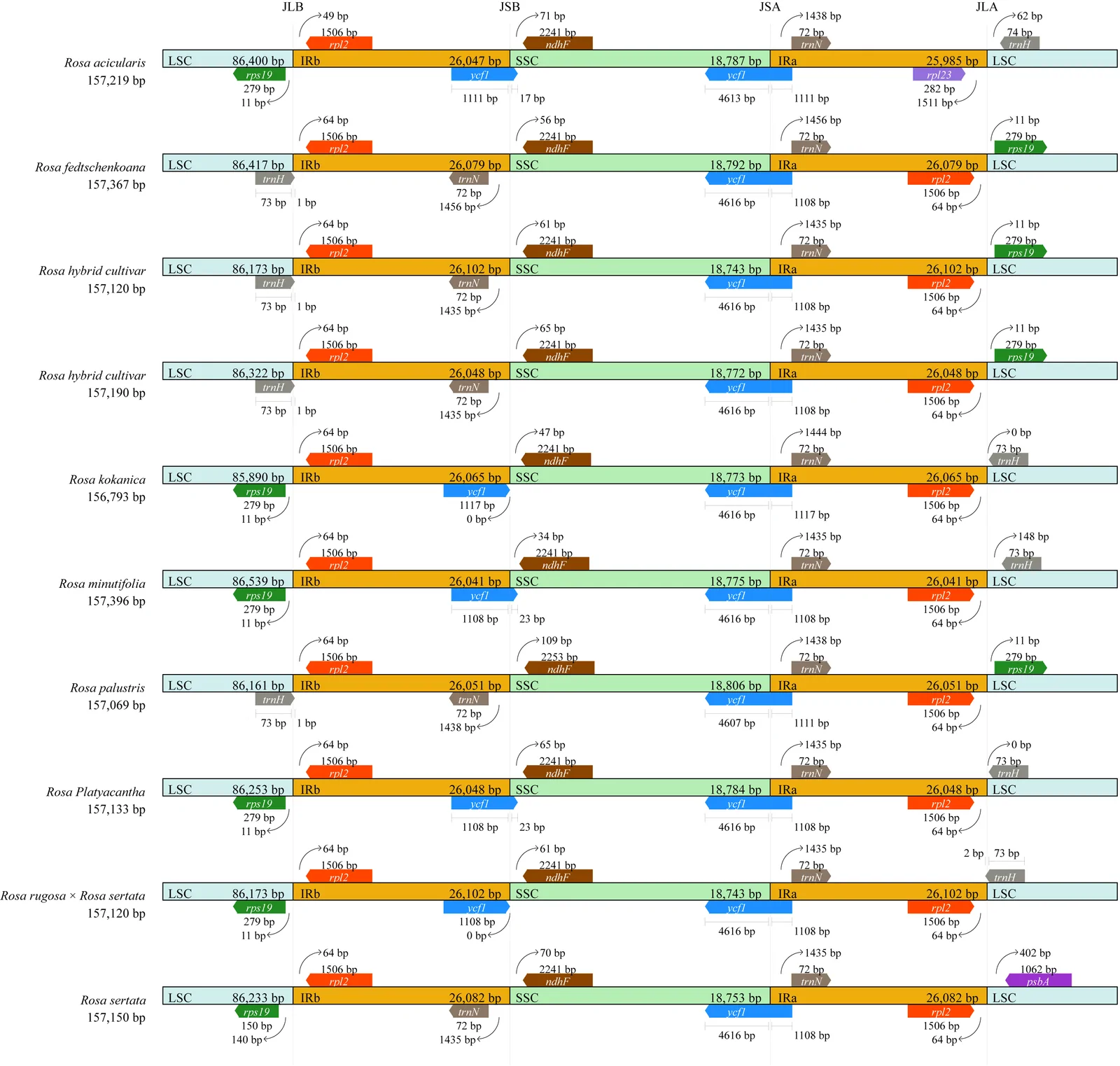
Comparison of boundary regions of chloroplast genomes in *Rosa*.

Analysis of IR region boundaries indicates that IR boundary characteristics are generally conserved across 10 *Rosa* species (Fig. 6). In most species, the lengths of IRa and IRb are consistent; only *R. acicularis* exhibits a difference of 62 bp: IRb: 26,047 bp; IRa: 25,985 bp. Among the boundary-associated genes at the LSC-IRb, IRb-SSC, SSC-IRa and IRa-LSC junctions, the *rps19* gene in the LSC region (279 bp) and the *rpl2* gene in the IRb region (1,506 bp) are conserved across all species without crossing the boundaries. The fragment length associated with the SSC boundary repeat gene ycf1 varied interspecifically between 1,108 and 1,117 bp. The *trnN* gene in the IRa region (72 bp) and the *trnH* gene in the LSC region (73–74 bp) were stably distributed near the LSC-IR boundary without transboundary migration. Only *R. acicularis* exhibited a 74 bp trnH gene, while all the others measured 73 bp.

### Phylogeny analysis of chloroplast genomes

**Figure 7:**
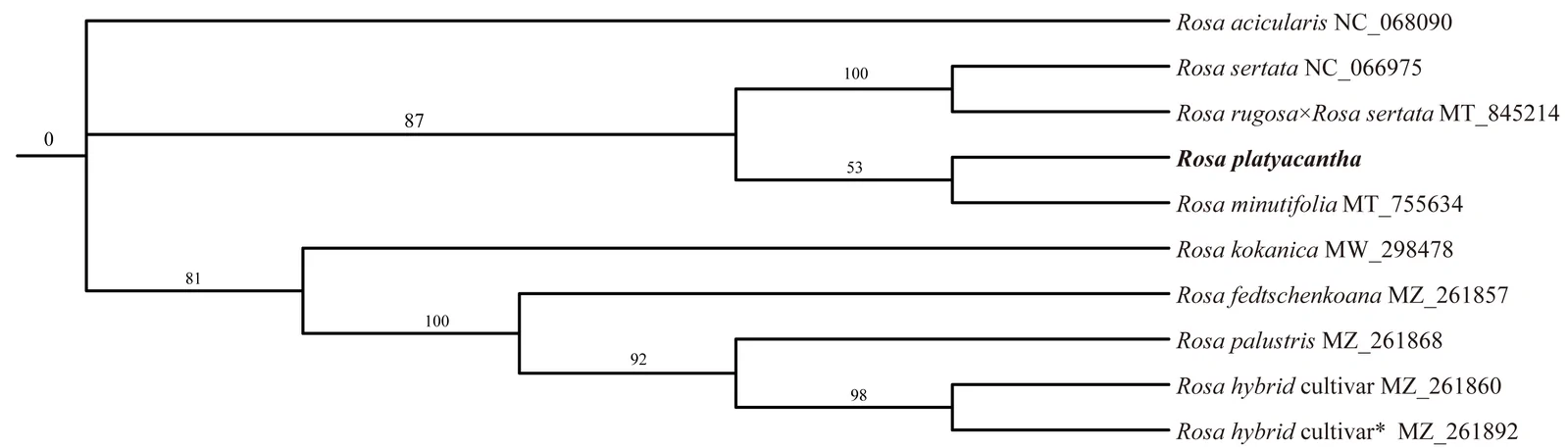
Phylogeny tree constructed based on the complete chloroplast genome of *Rosa*.

A phylogeny analysis of chloroplast genomes from 11 related plant species was conducted using the maximum likelihood method to determine the phylogeny relationships between *R. platyacantha* and other closely related species (Fig. 7). The results revealed five distinct nodes on the phylogeny tree. Starting from the first node, the eleven species were divided into three major evolutionary branches: RA formed a single branch; RS, RR, RPL and RM formed a single branch; and RK, RF, RP, RH1 and RH2 formed a single branch. Within the second clade, the support rate for the RS and RR hybrid branch was 100%. In contrast, the support rate for the RPL and RM branch was only 53%, suggesting a more distant relationship between RPL and RM. However, when these two branches were grouped together, the support rate increased to 87%. Nevertheless, the overall support rate for the aggregation of these three branches is 0%, suggesting relatively distant relationships among them and indicating that more closely related species exist within each branch. Notably, *Rosa* latifolia and the sympatric *R. fedtschenkoana* do not cluster into a single branch.

## Discussion

Wild populations of *Rosa* species constitute valuable plant resources, possessing significant medicinal potential. Given the extreme complexity of biological morphological characteristics within *Rosa* species, distinguishing between species and varieties proves challenging. To ensure the consistency of wild germplasm sources, comparative studies on genotypic differences between *Rosa chinensis* and closely related species within the genius have been conducted. These investigations provide crucial evidence for their taxonomic position within *Rosa* and elucidate adaptive evolutionary mechanisms within northwestern habitats.

This study fully sequenced the 157,133-base-pair chloroplast genome of *R. platyacantha*, which exhibits a typical tetradic circular structure. A total of 132 genes were annotated, with an overall GC content of 37.21%. These characteristics are highly consistent with those of most previously reported *Rosa* species (Zhou et al., 2025), reflecting the high conservation of chloroplast genomes within the genus *Rosa* (Gao et al., 2023). Notably, the *atpF* gene has three copies in *R. platyacantha*, while the *infA* gene is absent in both *R. platyacantha* and *R. majalis*. Such variations in gene copy number and loss are uncommon within *Rosa* and are potentially linked to specific metabolic regulation or evolutionary history, warranting further investigation. A total of 51 simple sequence repeat (SSR) loci were identified in *R. platyacantha*, with mononucleotide repeats dominating at 92.16%, predominantly of the A/T type. This composition is consistent with SSR characteristics observed in the chloroplast genomes of various plant families, including Moraceae and Rosaceae (Liu et al., 2025; Yang et al., 2022). SSR sequences play crucial functional roles in plant genomes, typically being closely associated with gene regulation, genome stability, and adaptive evolution. SSRs are ideal for developing high-resolution molecular markers, which facilitate in-depth studies of germplasm resource characterisation of *R. platyacantha* (Assunçao et al., 2025).

The codon usage in the chloroplast genome of *R. platyacantha* shows a clear preference for synonymous codons that end in A/U. Of the 29 preferred codons (RSCU > 1), 93.10% terminate in A/U. This correlates with the chloroplast genome’s high overall AT content, which could enhance translational efficiency under the stressful conditions prevalent in northwestern regions. This could be a manifestation of adaptive evolution at the molecular level (Tenzin & Zhang, 2025). Comparative genome analysis revealed significantly higher nucleotide diversity (Pi) in the LSC and SSC regions of *R. platyacantha* than in the conserved IR region. The analysis also identified four highly variable hotspots within the LSC region, including *ycf3*-*trnS*-GCU (Pi = 0.29005). These regions may function as crucial regulatory elements involved in controlling gene expression, thereby influencing the adaptation of *R. platyacantha* to environmental stressors (Shaw et al., 2014). Such hypervariable regions typically endure lower selective pressure, and their elevated mutation rates make ideal candidate fragments for investigating genetic differentiation among closely related species, species identification and adaptive evolution studies (Zhou et al., 2024a). This discovery provides precise targets for the subsequent in-depth exploration of key genes in the adaptive evolution of *Rosa* species. While the overall structure remains relatively conserved within the genus, analysis of the IR region boundaries revealed interspecific variation in the length of the *ycf1* gene segment at the boundary position. This provides clues at the structural level for understanding the evolutionary dynamics of the chloroplast genome in *Rosa* (Zhang et al., 2022).

Phylogeny analysis clearly shows that *Rosa* latifolia does not cluster with the sympatric *R. fedtschenkoana*. This finding confirms that the two species are distantly related at the genome level, providing new insights into the evolutionary and dispersal pathways of *Rosa* species within the unique habitats of north-west China. However, support for certain branches remains low, which is potentially due to the limited number of species included or weak phylogeny signals in specific regions of the chloroplast genome. Future studies that incorporate nuclear gene data or expand the scope of sampling hold promise for constructing a more robust phylogeny framework for *Rosa* (Cheng et al., 2025).

## Conclusions

The chloroplast genome of *R. platyacantha* spans 157,133 bp and exhibits a typical quadruple-stranded circular structure. It contains 132 annotated genes, with an overall GC content of 37.21%. While the genome structure shows high conservation within the genus *Rosa*, there are species-specific variations, such as the presence of three copies of the *atpF* gene and the absence of the *infA* gene. The genome exhibits a marked preference for synonymous codons ending in A/U, which could enhance translational efficiency under stressful conditions. Additionally, 51 simple sequence repeat (SSR) loci were identified, predominantly A/T-type mononucleotide repeats. These provide valuable resources for developing high-resolution molecular markers and for conducting population genetics and phylogeographic studies. Comparative genome analysis identified four highly variable hot spots within the LSC region, including *ycf3*-*trnS*-GCU (with Pi values as high as 0.29005). As potential adaptive-related loci, these highly variable regions provide precise targets for in-depth analysis of stress adaptation mechanisms in *Rosa* species. Phylogeny analysis definitively excluded *R. platyacantha* from clustering with the sympatric *R. fedtschenkoana*, providing novel molecular evidence to elucidate evolutionary pathways within *Rosa* in the unique northwestern habitat. This study fills the gap in chloroplast genome information for *R. platyacantha* and provides crucial genome evidence for analysing its habitat adaptation potential through multidimensional comparative and evolutionary analyses. The study lays a theoretical foundation for the scientific conservation and sustainable utilisation of this endemic germplasm resource.

##  Supplemental Information

10.7717/peerj.21639/supp-1 Supplemental Information 1 Genome Sequence Information for *R. platyacantha .*

10.7717/peerj.21639/supp-2 Supplemental Information 2 Genome Sequence GenBank for R. platyacantha

10.7717/peerj.21639/supp-3 Supplemental Information 3 Raw data for all experiments on the complete chloroplast genome sequence of Rosa platyacantha The raw data on which all the figures in this study are based. It comprises the following worksheets: (1) complete chloroplast genome sequences for 10 species of the genus Rosa; (2) a codon usage table for protein-coding genes; (3) identified simple sequence repeats (SSRs), including their patterns and loci.

## Additional Information and Declarations

### Data Availability

The following information was supplied regarding data availability: The data is available in the Supplemental Files. The raw sequence data are available at GenBank: PZ127297.
